# Resting-State Neural Firing Rate Is Linked to Cardiac-Cycle Duration in the Human Cingulate and Parahippocampal Cortices

**DOI:** 10.1523/JNEUROSCI.2291-18.2019

**Published:** 2019-05-08

**Authors:** Kayeon Kim, Josef Ladenbauer, Mariana Babo-Rebelo, Anne Buot, Katia Lehongre, Claude Adam, Dominique Hasboun, Virginie Lambrecq, Vincent Navarro, Srdjan Ostojic, Catherine Tallon-Baudry

**Affiliations:** ^1^Laboratoire de Neurosciences Cognitives et Computationnelles, Institut National de la Santé et de la Recherche Médicale, École Normale Supérieure, Paris Sciences et Lettres Research University, 75005 Paris, France,; ^2^Institut du Cerveau et de la Moelle épinière, Institut National de la Santé et de la Recherche Médicale, Sorbonne Université, 75252 Paris, France, and; ^3^Epileptology Unit and Neurophysiology Department, Hôpitaux Universitaires Pitié Salpêtrière Charles Foix, 75013 Paris, France

**Keywords:** heart rate, human cortex, resting state, spontaneous neural firing

## Abstract

Stimulation and functional imaging studies have revealed the existence of a large network of cortical regions involved in the regulation of heart rate. However, very little is known about the link between cortical neural firing and cardiac-cycle duration (CCD). Here, we analyze single-unit and multiunit data obtained in humans at rest, and show that firing rate covaries with CCD in 16.7% of the sample (25 of 150). The link between firing rate and CCD was most prevalent in the anterior medial temporal lobe (entorhinal and perirhinal cortices, anterior hippocampus, and amygdala), where 36% (18 of 50) of the units show the effect, and to a lesser extent in the mid-to-anterior cingulate cortex (11.1%, 5 of 45). The variance in firing rate explained by CCD ranged from 0.5 to 11%. Several lines of analysis indicate that neural firing influences CCD, rather than the other way around, and that neural firing affects CCD through vagally mediated mechanisms in most cases. These results show that part of the spontaneous fluctuations in firing rate can be attributed to the cortical control of the cardiac cycle. The fine tuning of the regulation of CCD represents a novel physiological factor accounting for spontaneous variance in firing rate. It remains to be determined whether the “noise” introduced in firing rate by the regulation of CCD is detrimental or beneficial to the cognitive information processing carried out in the parahippocampal and cingulate regions.

**SIGNIFICANCE STATEMENT** Fluctuations in heart rate are known to be under the control of cortical structures, but spontaneous fluctuations in cortical firing rate, or “noise,” have seldom been related to heart rate. Here, we analyze unit activity in humans at rest and show that spontaneous fluctuations in neural firing in the medial temporal lobe, as well as in the mid-to-anterior cingulate cortex, influence heart rate. This phenomenon was particularly pronounced in the entorhinal and perirhinal cortices, where it could be observed in one of three neurons. Our results show that part of spontaneous firing rate variability in regions best known for their cognitive role in spatial navigation and memory corresponds to precise physiological regulations.

## Introduction

What does the brain at rest do? While there is no simple answer to this question, it is usually agreed that the spatiotemporal structure of spontaneous brain activity is meaningful (Arieli et al., 1996; Berkes et al., 2011) and that it can influence responses to stimuli (Greicius et al., 2004). However, the brain at rest is not only preparing to respond to future stimuli; it is also constantly engaged in monitoring and regulating bodily organs, such as the heart.

Central brain regions engaged in heart-rate regulation include large portions of the cingulate cortex, the ventromedial prefrontal cortex, the insula, and the amygdalohippocampal formation. The role of those regions in heart-rate regulation was established by a combination of anatomical tracing, stimulation, and lesion studies in rats (Cechetto and Saper, 1990; Benarroch, 1993; Verberne and Owens, 1998; Westerhaus and Loewy, 2001), cats, and monkeys (Anand and Dua, 1956). There is good agreement about the areas involved in heart-rate regulation as identified in humans by direct electrical stimulation in patients (Pool and Ransohoff, 1949; Stock et al., 1978; Oppenheimer et al., 1992; Parvizi et al., 2013) or with functional magnetic resonance imaging during tasks (for review, see Cechetto and Shoemaker, 2009; Thayer et al., 2012; Beissner et al., 2013; Gianaros and Wager, 2015) or at rest (Chang et al., 2013; Rebollo et al., 2018). This suggests that neural firing might be directly related to heart-rate regulation, at least in so-called limbic regions.

Surprisingly, the literature on neural variability at the single-neuron level contains very little about the link between neural activity and heart rate. However, the link between local field potentials and respiration has recently been examined (Tort et al., 2018). Spontaneous variations in firing rate are most often attributed to cellular machinery noise (Faisal et al., 2008), or to brain state (Poulet and Petersen, 2008; Deco and Hugues, 2012; McGinley et al., 2015) and fluctuations in excitability (Goris et al., 2014) related to top-down factors (Gilbert and Sigman, 2007). However, some of the ongoing fluctuations in cortical firing rate might be related to heart rate. Indeed, two reports that have received little notice describe a relation between the duration of the cardiac cycle and firing rate in the cat somatosensory thalamus (Massimini et al., 2000), as well as in the human amygdala and hippocampus (Frysinger and Harper, 1989, 1990), i.e., in structures that interact massively with cortical regions.

Here, we directly investigated the link between cardiac-cycle duration (CCD) and spontaneous neural firing rate in single-unit and multiunit activity (SUA and MUA) recorded in humans at rest. Recording sites were determined for diagnostic and therapeutic purposes only, but happened to be often located in regions known to be related to heart rate, i.e., in the medial temporal lobe (MTL; parahippocampal gyrus, hippocampus, and amygdala) and in the cingulate cortex.

**Figure 1.**
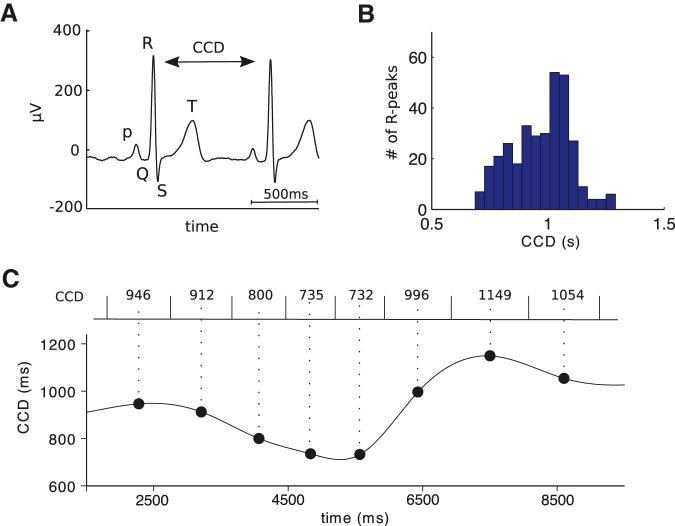
Measuring the cardiac cycle. ***A***, An example ECG trace showing the QRS complex. CCD is defined as the time between two successive R peaks. ***B***, Histogram of CCD distribution in one patient. ***C***, The CCD time series (bottom) is constructed by assigning each CCD (top) to the central time point (large black dots) between two heartbeats, then interpolating the points with a cubic spline function.

Before presenting the results, we remind readers of some basic facts about the heart rate and its central regulation. The CCD, which can be quantified with electrocardiograms (ECGs), varies spontaneously during the resting state (Fig. 1). The CNS can affect CCD (Somsen et al., 2004; Berntson et al., 2007; Thayer et al., 2012; Gianaros and Wager, 2015) by modulating the vagal output to the cardiac pacemaker, resulting in fast, beat-to-beat, but also longer-lasting changes in heart rate. Sympathetic influences affect heart-rate fluctuations with a delay of several seconds and induce long-lasting changes, via the modulation of cardiac contractibility and vessel resistance.

Following the approach used in Frysinger and Harper (1989) and Massimini et al. (2000), we first examined the correlation between neuronal firing rate and CCD, and further characterized the firing properties of the neurons that exhibit a strong correlation. To provide indications on the underlying mechanisms, we then investigated the temporal aspects of the link between firing rate and CCD. Last, we analyzed the directionality of interactions from the neuron to the heart and from the heart to the neuron.

## Materials and Methods

### 

#### 

##### Subjects.

Twelve patients with pharmacoresistant focal epilepsy (five male; seven female; mean age, 31.9 ± 8.83 years) were stereotactically implanted with depth electrodes to determine the seizure-onset zone for potential surgical resection. Implantation sites were selected for diagnostic and therapeutic purposes only. During the following seizure monitoring period, participants provided informed and written consent to participate in the study. At the time of recordings, all patients received at least one medication that could interfere with cardiac activity (most often, carbamazepine, lamotrigine, or lacosamide; Table 1). All procedures were approved by the local ethics committee (Comités de Protection des Personnes Paris VI, Institut National de la Santé et de la Recherche Médicale C11-16).

**Table 1.** Patients and recording sites

Patient number	Age (years)	Gender	Medication	Heart rate (beats/min)	MNI mm (*x*, *y*, *z*)	Anatomical description	Number of units	Cardiac-cycle effect*^a^*	Contact in seizure-onset zone
1	30	Male	Oxcarbazepine, lacosamide, clobazam	61	−24, −3, −30	Parahippocampal gyrus	8	1 R−	No
					−38, −29, −23	Parahippocampal gyrus	2	0	No
2	26	Male	Carbamazepine, valproate	68	30, −5, −27	Parahippocampal gyrus	1	0	No
					41, −54, −15	Fusiform gyrus	6	0	No
3	23	Female	Carbamazepine, topiramate, lacosamide	80	−25, −14, −16	Parahippocampal gyrus	5	2 R+	No
					−20, −20, −12	Parahippocampal gyrus	2	0	No
4	32	Male	Levetiracetam, lamotrigine, carbamazepine	76	−17, −5, −21	Amygdala/parahippocampal gyrus	7	1 R+, 2 R−	Yes
					−18, −14, −21	Hippocampus	9	0	No
5	22	Female	Valproate, lamotrigine	88	5, −4, 49	Midcingulate sulcus	8	0	No
					10, 20, 27	Anterior cingulate sulcus	13	1 R−	Yes
6	40	Female	Lacosamide, phenytoine, ventoline,	71	−16, −10, −26	Hippocampus/parahippocampal gyrus	6	0	No
			Piasclédine		−9, 38, −20	Subgenual anterior cingulate	2	0	No
					−27, −7, −46	Parahippocampal gyrus	7	1 R+	Yes
7	48	Female	Lamotrigine, zonisamide, perampanel	92	−20, −2, −31	Parahippocampal gyrus	7	2 R+	Yes
					−24, −20, −16	Hippocampus/parahippocampal gyrus	3	0	Yes
8	34	Male	Carbamazepine, lamotrigine	65	24, −14, −26	Hippocampus/parahippocampal gyrus	11	0	No
					21, −5, −39	Parahippocampal gyrus	7	0	No
9	26	Female	Levetiracetam, lacosamide, valproate, fluoxetine, zolpidem, diazepam, propanolol	63	22, −3, −34	Parahippocampal gyrus	13	11 R−	Unknown*^b^*
10	25	Female	Lamotrigine, lacosamide, clobazam	68	10, 18, 30	Anterior cingulate gyrus	7	0	No
11	47	Male	Eslicarbazepine, zonisamide	69	−18, −65, −10	Fusiform gyrus	9	0	No
12	30	Female	Lamotrigine, levetiracetam, carbamazepine, perampanel	69	−2, 30, 21	Anterior cingulate gyrus	7	1 R+	No
					−2, 5, 41	Midcingulate gyrus	10	1 R+, 2 R−	No

*^a^*R+: unit showing a positive correlation between firing rate and cardiac cycle duration (*p* < 0.05, FDR corrected); R−: unit showing a negative correlation.

*^b^*Premature interruption of the clinical procedure; no seizure recorded.

##### Recordings.

The depth electrodes (AdTech, Behnke-Fried type) consisted of eight macrocontacts (platinum; diameter, 1.3 mm) embedded on the surface of a polyurethane tube with a hollow lumen. Eight 40-μm-diameter platinum microwires, including one used as a reference, protruded 3–6 mm into the cerebral tissue beyond the tip of the deepest macrocontact. The ECG was recorded from two cutaneous electrodes on the upper chest, but the exact positioning was not standardized. All signals were collected using a 160-channel recording system (Neuralynx, Atlas) with a 32 kHz sampling rate and a 0.1–8000 Hz bandpass filter for microwire data and a 4 kHz sampling rate and 0.1–1000 Hz bandpass filter for the ECG.

##### Experimental design.

During data collection, seated subjects fixated a central point on a gray background displayed on a laptop monitor. The extracellular neural signals analyzed in the current study came from either one continuous recording during resting-state fixation lasting 5–6 min, or from shorter blocks of 13–30 s of resting-state fixation embedded within a task (Babo-Rebelo et al., 2016).

##### Electrode localization.

Anatomical localization was based on individual postoperative MRI warped to the MNI brain using the EpiLoc toolbox developed on the STIM platform (stereotaxy, techniques, images, models; http://pf-stim.cricm.upmc.fr), as well as on the computed tomography scan aligned with MRI data. Note that the microelectrode bundle opens at the tip of the macroelectrode, with a distance of a few millimeters between microwires. The localization described in Table 1 provides an anatomical description of all eight microwires, which in some cases could span different regions.

##### Spike detection and spike time series.

An adaptive filtering procedure (Keshtkaran and Yang, 2014) was applied to the raw data to limit power-line interference as well as harmonics. Spike detection and waveform sorting were performed using the semiautomatic procedure implemented in the software wave_clus (Quiroga et al., 2004). Data were first bandpass filtered between 300 and 3000 Hz (elliptic bandpass filter, fourth order) and spikes were detected using the automatic amplitude threshold algorithm. Thresholds for spike detection ranged between 3.5 and 5.5 SDs. Waveforms were then clustered according to the superparamagnetic clustering algorithm. Typically, one to three clusters were isolated from each microelectrode where spikes had been identified. A number of additional steps were applied for selecting valid units after cluster isolation.

First, we observed waveforms for each cluster and discarded clusters displaying multiple peaks (*n* = 5). Second, we discarded clusters without any spike for ≥45 consecutive cardiac cycles (*n* = 7). We then removed the five clusters with the lowest firing rate (<0.2 spikes/s). All remaining clusters (150) were considered valid units. Units were further identified as single or multiunit based on the percentage of very short interspike intervals (ISIs). Units with <1% of ISIs <3 ms were classified as single units. All others were classified as multiunits. After applying all the steps described above, 108 single units and 42 multiunits were selected for further analysis.

Spike times were downsampled from 32 to 1 kHz. Spike density, a continuous estimate of instantaneous firing rate, was estimated by convoluting the spike time series with a Gaussian kernel (20 ms SD). The spike-density time series were used for the spectral analysis and for the computation of coherence with heart-rate variability (HRV), as well as to search for transient changes in firing rate in response to heartbeats.

##### CCD and CCD time series.

We detected R peaks in the ECG using a semiautomatic procedure that involved correlating the ECG with a template QRS complex defined on a subject-by-subject basis, and the manual verification of all R peaks separated from their neighbors by very short or very long intervals. R-peak timings were downsampled from 4 to 1 kHz. CCD, or interbeat interval, was defined as the latency difference between two successive R peaks (Fig. 1*A,B*). In the four patients with ≥5 min of continuous recordings, CCD time series were created by assigning each CCD to the central time point between the heartbeats corresponding to the cardiac cycle, and interpolating with a cubic spline function (Fig. 1*C*). These time series were used for the spectral analysis of HRV as well as for cross-correlation between the ISI and CCD time series.

##### Linear correlation analysis between firing rate and CCD.

To quantify the correlation between firing rate and CCD in each unit, we computed the mean firing rate at each cardiac cycle (number of spikes during a cardiac cycle divided by cycle duration, spike/s) and computed for each unit the Pearson correlation coefficient between the firing rate and CCD, across all cardiac cycles. Statistical significance was evaluated using a permutation-based procedure, where the original order of CCDs was shuffled 10,000 times. The resulting Monte-Carlo *p* values were corrected for multiple comparisons across recording sites using the false discovery rate (FDR) procedure (Hochberg and Benjamini, 1990).

##### Spectral analysis of HRV and spike density.

We analyzed both HRV and spike-density time series in the frequency domain in the four patients in whom we recorded 5 min of continuous data. Spectral analysis was performed using the FieldTrip toolbox for Matlab (Oostenveld et al., 2011), resulting in a multitaper frequency transformation in sliding 60 s time windows with 6 s overlap. Coherence between the HRV and spike-density spectral estimates was computed using the FieldTrip function ft_connectivityanalysis.m.

##### Cross-correlation and coupling between inter spike interval (ISI) and CCD time series.

To assess whether the fluctuations of neuronal spiking and heartbeat activity are correlated, we computed cross-correlograms between a smoothed neuronal ISI and CCD time series. Created in a manner similar to that used to produce a CCD time series, a neuronal ISI time series was generated by assigning to each ISI center time point the ISI duration. Then, interpolation was applied using cubic splines. We estimated the coupling kernel, α(*t*), which expresses the systematic interaction between the two smoothed time series over the whole recording duration: CCD(*t*) = ∑*l*α(l) · ISI(*t* – *l*). Specifically, we calculated this kernel using (inverse) Fourier transforms, α(*t*) = ifft{fft(CCD(*t*))/fft(ISI(*t*))}.

Computations were performed for each continuous recording block. Results were averaged across the available blocks. The contribution of each block was weighted by its duration.

##### Detection of transient changes in instantaneous firing rate in response to heartbeats.

Data were epoched from one R peak of the ECG to the next. Data analysis was performed by analyzing the data locked to the onset of the epoch, by analyzing the data locked to the end of the epoch, or by normalizing epoch duration from the current heartbeat to the next, latencies being then expressed in percentage of CCD. Results were similar in all three cases and the results reported here correspond to the normalized epoch duration. The existence of transient increases or decreases in firing rate in the spike-density time series was then assessed by a cluster-based permutation procedure (Maris and Oostenveld, 2007) that intrinsically corrects for multiple comparisons. For each unit, we created 10,000 surrogate datasets where R-peak latencies were shuffled, but the CCD distribution was preserved. We compared at each time point the observed spike-density value with the distribution of the 10,000 surrogate spike-density value to derive a Monte-Carlo *p* value. Adjacent time points with a Monte-Carlo *p* value <0.01 defined candidate clusters. Monte-Carlo *p* values were then converted into *z* scores. In a second step, we summed over time the *z* scores of each candidate cluster. We repeated this procedure on the 10,000 surrogate datasets, retaining for each surrogate dataset the largest *z* sum, to obtain the maximum (*z* sum) distribution that could be obtained under the null hypothesis. The comparison between the observed *z* sum and distribution of surrogate *z* sum identifies clusters of significantly increased or decreased firing in the original data, with a Monte-Carlo *p* < 0.05, two-tailed. The resulting Monte-Carlo *p* values were corrected for multiple comparisons across recording sites using the FDR procedure (Hochberg and Benjamini, 1990).

##### Phase–response analysis.

To quantify the effect of occurrence and timing of spikes on CCD, we calculated phase–response curves (PRCs) using a method adopted from Blot et al. (2016; where these curves are referred to as delayed spike curves due to the specific context). The PRC measures how much a spike within a cardiac cycle shortens or lengthens the current CCD on average. In particular, defining *T*_−_ as the time duration since the previous heartbeat and *T*_+_(*T*_−_) as the time duration to the next heartbeat (which naturally depends on *T*_−_), the PRC is expressed as the averaged difference of the duration *T*_+_(*T*_−_) observed (using heartbeat and spike times) and the duration 〈*T*_+_(*T*_−_)〉_exp_ that is expected based on knowledge of the CCD distribution only: PRC(*T*_−_) = <*T*_+_(*T*_−_) − <*T*_+_(*T*_−_)>_exp_>, where the latter term is calculated by

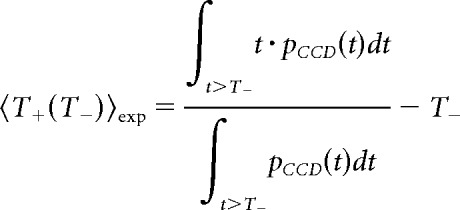
 and the (outer) average < > is calculated using a suitable binning of CCDs. We considered two types of binning: only one bin corresponding to the whole cardiac cycle, and two bins of different lengths corresponding to systole and diastole. Systole duration was determined in each patient according to the formula proposed by Fridericia (2003):

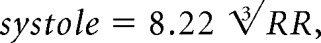
 where RR is the mean CCD of the patient. Diastole was defined, at each cardiac cycle, as the remaining part of the cardiac cycle. Note that this method cannot easily be extended to the analysis of adjacent cycles in a meaningful way.

To test for significance, we applied a permutation-based procedure using a large number (10,000) of surrogate spike trains generated by shuffling the ISI of the original data. We used the Hochberg and Benjamini procedure (Hochberg and Benjamini, 1990) to control for multiple comparisons across recording sites by maintaining the FDR at *p* < 0.05.

PRC values computed over one cardiac cycle are positive in cells showing a positive correlation between firing rate and CCD (R+ cells) and negative in R− cells. Considering the two-bin (systole, diastole) PRC, we tested for the existence of a significant reduction in PRC from systole to diastole (i.e., positive or negative values getting closer to zero) by multiplying systolic and diastolic PRC values in R− cells by −1, and comparing the resulting PRC values in systole and diastole using a two-tailed paired *t* test.

##### Statistical analysis.

As described above, all statistical analysis relied first on the estimation of a Monte-Carlo *p* value at each recording site, obtained by comparing the empirical result with the distribution under the null hypothesis computed on data in which CCDs have been shuffled, and then on a control for multiple comparisons across recording sites using the Hochberg and Benjamini procedure (Hochberg and Benjamini, 1990) to maintain the FDR at *p* < 0.05.

Bayes factors were computed using the online tool (http://pcl.missouri.edu/bayesfactor), which is based on Liang et al. (2008). Here, a Bayes factor >10 indicates strong evidence for the null hypothesis, between 3.2 and 10 indicates substantial evidence for the null hypothesis, and <3.2 indicates inconclusive evidence for or against the null hypothesis (Kass and Raftery, 1995).

##### Code accessibility.

Matlab/Python code is freely available at https://github.com/neuromethods/neural-firing-and-cardiac-cycle-duration.

## Results

### Data summary

**Figure 2.**
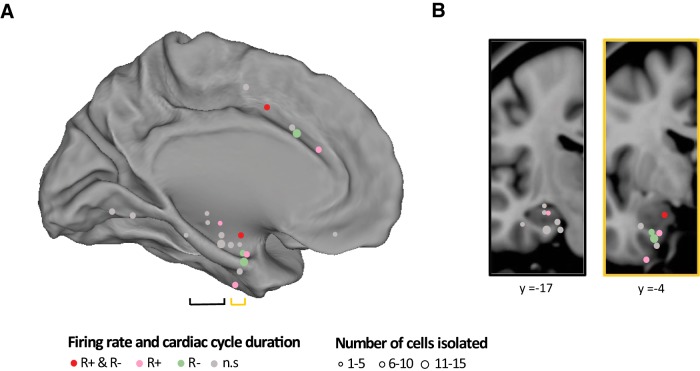
Anatomical description of recording sites. ***A***, All 22 recording sites from 12 patients projected on the medial view of an inflated hemisphere, with left and right hemispheres collapsed for visualization purposes. Each recording site is represented by a dot, with size indicating the number of cells isolated. Dot color indicates sites with at least one unit with a significant increase in firing rate for slower heart rate (positive correlation between firing rate and CCD, pink), with at least one unit with a significant decrease in firing rate for slower heart rate (negative correlation, green), or both (red). Sites where no unit showed a significant link between firing rate and heart rate are presented in gray. ***B***, Coronal brain views with a projection of medial temporal recording sites with *y* comprised between −10 and −30 (black contour, left) and sites with *y* comprised between −2 and −7 (yellow contour, right), corresponding to the black and yellow brackets in ***A***.

We recorded unit activity together with the ECG in 12 epileptic patients (Table 1) with normal heart rate (mean ± SEM: 72.5 ± 2.8 beats/min; range, 61.4–91.8) during passive fixation. We isolated 150 units (108 SUAs, 42 MUAs), recorded from 112 microelectrodes at 22 recording sites. The average firing rate across all units was (mean ± SEM) 2.54 ± 0.10 spikes/s (SUA, 2.28 ± 0.09; MUA, 3.19 ± 0.12 spikes/s). Fourteen recording sites (Fig. 2*A*), corresponding to 88 units, were located in the MTL (parahippocampal gyrus, hippocampus, and amygdala). Five recording sites (45 units) were located in the midcingulate and anterior cingulate regions while the ventral visual pathway (two recording sites in the fusiform gyrus, 15 units) or the subgenual anterior cingulate cortex (one recording site, two units) were only occasionally sampled.

### Correlation between spontaneous firing rate and CCD

**Figure 3.**
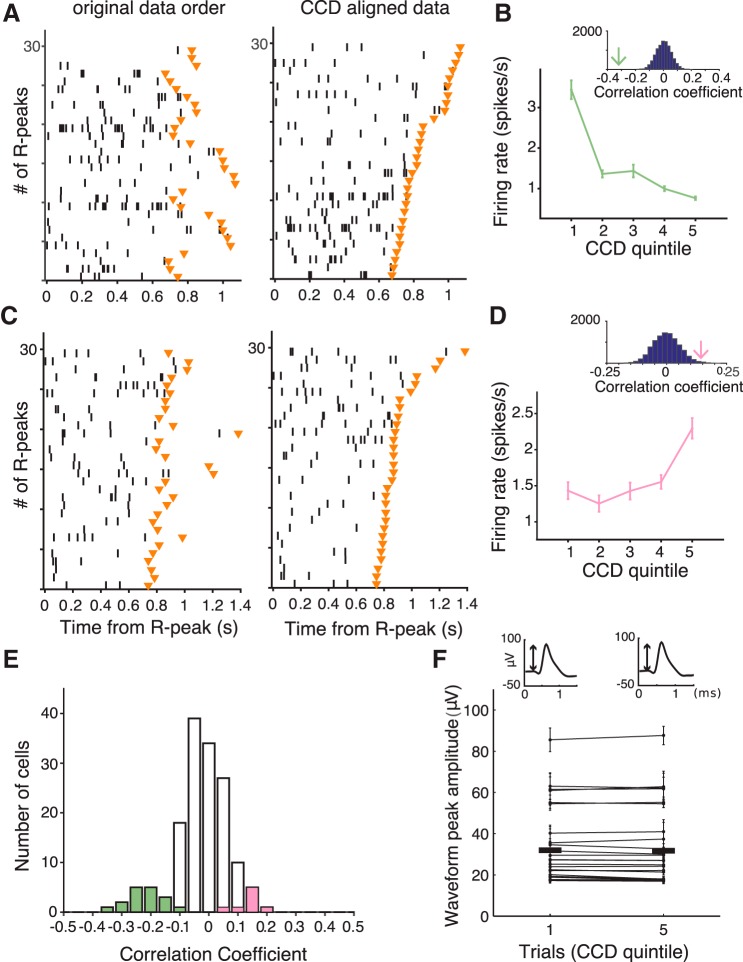
Correlation between firing rate and CCD. ***A***, Example cell (#128, patient #9, parahippocampal gyrus) with a negative correlation between firing rate (spikes/s) and CCD. The left panel is the spike raster plot where the *y*-axis corresponds to 30 consecutive cardiac cycles, organized by order of occurrence from first (bottom) to last (top), and the *x*-axis corresponds to the time from current R peak to the next R peak marked by the orange triangle. The middle panel corresponds to the same raster as the left one, but the data are sorted according to increasing CCDs, from short (bottom) to long (top) cardiac cycles. ***B***, Mean firing rate (±SEM) as a function of CCD duration (quintiles, from short to long CCD), in the same cell as in ***A***, for the full dataset (365 cardiac cycles). The inset illustrates the distribution of correlation coefficients between firing rate and CCD derived from surrogate data. The green arrow indicates the correlation coefficient of the empirical data. ***C***, ***D***, Example cell (#162, patient #12, anterior cingulate), showing a positive correlation between firing rate and CCD. Conventions are the same as in ***A*** and ***B***. ***E***, Population histogram (*n* = 150 units) of the correlation coefficient between firing rate and CCD. The colored bars indicate cells with significant positive (pink) and negative (green) correlation between firing rate and CCD (FDR corrected, *p* < 0.05). ***F***, Comparison of spike waveform amplitude peak between first and last CCD in quintile (*n* = 25). Horizontal black bars indicate mean of waveform peak amplitude within first and last CCD in quintile over 25 significant units. The top two insets show example waveforms averaged over first and last CCD in quintile. The peak amplitude is indicated by the arrow.

Like Frysinger and Harper (1989) and Massimini et al. (2000), we first computed the mean firing rate during each cardiac cycle, and observed a correlation between spontaneous firing rate and CCD. Figure 3*A*,*B* shows an example of a cell in the parahippocampal gyrus whose spontaneous firing rate is negatively correlated with CCD. Figure 3*C*,*D* shows an example of a cell in the anterior cingulate gyrus whose firing rate is positively correlated with CCD.

Across all units, the correlation coefficient shows a bias toward negative values (Fig. 3*E*; mean Pearson *r* = −0.027 ± 0.008, *t* test against 0 on Fischer-*Z*-transformed correlation coefficients, *t*_(149)_= −3.51, *p* < 10^−3^). This result indicates that at the population level, the firing rate is higher when the heart is beating faster. However, this effect was not evenly distributed among units.

We therefore tested the significance of the correlation between firing rate and CCD at the level of each individual unit. We found that 25 of 150 units (16.7%, from eight different patients and nine recording sites) showed a significant correlation between firing rate and CCD (Fig. 3*E*; Pearson correlation, all FDR-corrected *p*'s < 0.05). In those 25 neurons, CCD explained on average 4.25 ± 0.53% of spontaneous firing-rate variance, ranging from 0.5 to 11%.

In 17 of these neurons (seven SUAs, 10 MUAs), the correlation was negative, i.e., elevated firing rates corresponded to shorter cardiac cycles (mean *r* = −0.22 ± 0.014) and in eight neurons (seven SUAs, one MUA), the correlation was positive, i.e., elevated firing rates corresponded to longer cardiac cycles (mean *r* = 0.14 ± 0.014). Those neurons will be hereafter referred to as R− and R+ neurons, respectively. The firing-rate variance explained by CCD was significantly larger for R− units (5.26 ± 0.62%) than for R+ units (2.1 ± 0.4%; Mann–Whitney *U* test *p* = 0.002).

### The correlation between spontaneous firing rate and CCD is most prominent in the anterior part of the MTL and cingulate cortex

The MTL was well represented in our dataset. In the MTL, 22.7% of the units (20 of 88) showed a significant correlation with CCD. The most anterior MTL regions, corresponding to the anterior parahippocampal cortex, a region that encompasses the entorhinal and perirhinal cortices, anterior hippocampus, and amygdala, seemed to be particularly involved (Fig. 2). To quantify the difference between posterior and anterior MTL regions, we labeled MTL regions anterior if *y* ≥ −7 and posterior if *y* ≤ −10. The anterior MTL regions contained the highest proportion of units (36%, 18 of 50 units) with a significant CCD effect (Fig. 2*B*; Table 1). This percentage fell to 5.3% (two units of 38) for posterior (*y* ≤ −10) MTL recording sites. In the midcingulate and anterior cingulate sites, 11.1% of the units showed a significant CCD effect (five of 45 units; proportion lower but not significantly different from that found in MTL, χ^2^ test, χ^2^ = 2.63, df = 1, *p* = 0.10). Last, no CCD effect was observed in regions with fewer samples (fusiform gyrus, zero of 15 units, two patients; subgenual anterior cingulate gyrus, zero of two units, one patient). All the contacts showing a significant correlation between firing rate and CCD broadly belong to the mid-to-anterior cingulate cortex, and to the amygdalohippocampal formation and adjacent cortices.

Positive and negative correlations between firing rate and CCD could be found in the same region (Fig. 2*A*) in different patients. In two instances, we even found positive and negative correlations at the same location in the same patient in neurons recorded by different microwires, i.e., separated by a few millimeters only (patient #4, amygdala/parahippocampal gyrus, one R+, two R−; patient #12, midcingulate cortex, one R+, two R−).

### Neurons showing a significant CCD effect are more variable

Does firing rate in neurons with a significant CCD effect differ from firing rate in other neurons? We first compared the firing rate in neurons with a significant CCD effect (*n* = 25; mean firing rate, 2.14 ± 1.28 spike/s) to the firing rate of all other neurons (*n* = 125; mean firing rate, 2.62 ± 2.21 spike/s), but found no significant difference [unpaired *t* test; *t*_(148)_ = −1.03; *p* = 0.30; Bayes factor (BF), 2.79; inconclusive]. The firing of R− neurons (2.13 ± 1.07 spike/s) was similar to that of R+ neurons (2.17 ± 1.74 spikes/s; unpaired *t* test; *t*_(23)_ = 0.08; *p* = 0.94; BF, 2.58; inconclusive).

**Figure 4.**
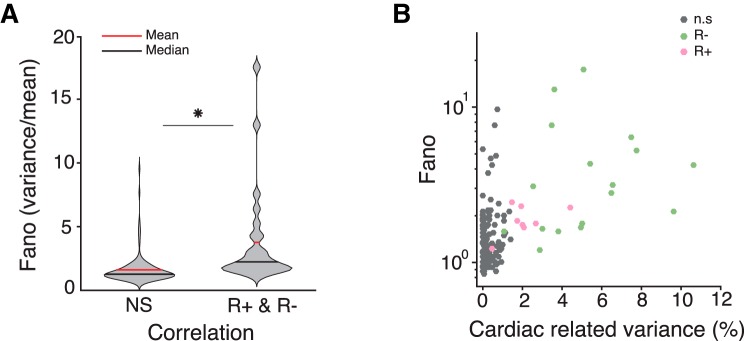
FF and cardiac-related variance. ***A***, Violin plot indicating mean (red) and median (black) of FF of units showing no significant correlation (NS) and units showing significant correlation with CCD (R+, R−). ***B***, The FF (on a log_10_ scale for graphical purpose only) is plotted as a function of variance in spontaneous firing rate explained by CCD, for units showing no significant correlation (gray), a negative correlation (green), and a positive correlation (pink) between firing rate and CCD. *p < 10^-6^.

We then computed a compact measure of firing variability, the Fano factor (FF; Fig. 4*A*), corresponding to the mean number of spikes divided by the variance in number of spikes, computed over 1 s time windows. The FF of neurons showing a significant CCD effect (*n* = 25; FF, 3.78 ± 3.90) was significantly larger than the FF of nonsignificant neurons (*n* = 125; FF, 1.62 ± 1.18; unpaired *t* test; *t*_(148)_ = 5.18; *p* < 10^−6^). The FF was increasing with cardiac-related variance (Fig. 4*B*), with a significant correlation between the two (*n* = 150, Pearson correlation, *r* = 0.51, *p* < 10^−9^).

### Controls on recording stability and epileptic activity

We examined whether the link between spontaneous spiking activity and CCD could be due to some cardiac-related recording instability. We tested whether the spike waveform differed depending on CCD, as proposed by Frysinger and Harper (1989). We measured waveform peak amplitude in spikes occurring in the 20% shortest versus the 20% longest cardiac cycles and tested for possible differences. The spike waveform was stable and did not depend on CCD in the 25 neurons showing a significant cardiac effect (Fig. 3*F*; waveform peak amplitude, paired *t* test between spikes occurring in short and long cardiac cycles, *t*_(24)_ = 0.043; *p* = 0.97; BF, 4.65; indicating substantial evidence for the null hypothesis) or across the 150 neurons tested (paired *t* test; *t*_(149)_ = 0.099; *p* = 0.92; BF, 10.9; indicating strong evidence for the null hypothesis). Thus, the relationship between spontaneous firing rate and CCD does not arise from cardiac-related recording instability.

We also verified that spike waveform peak amplitude was stable across recording time. The waveform peak amplitude in the first 20% of the recordings was not different from the last 20% in the 25 neurons showing significant CCD effect (paired *t* test; *t*_(24)_ = 0.13; *p* = 0.90; BF, 4.62; indicating substantial evidence for the null hypothesis) or in the 150 neurons analyzed (paired *t* test; *t*_(149)_ = 0.098; *p* = 0.92; BF, 10.91; indicating strong evidence for the null hypothesis).

Last, we verified that the correlation between firing rate and CCD was not confined to the seizure-onset zone, which could be determined for all but one patient (Table 1). Four of the nine recording sites showing the correlation were located outside the seizure-onset zone (two in the anterior parahippocampal cortex, two in the cingulate cortex).

### Temporal extension of the link between firing rate and CCD

**Figure 5.**
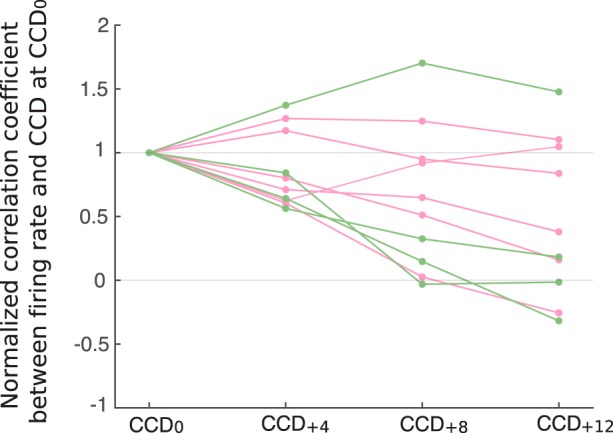
Correlation between firing rate and CCD over time. Firing rate at a given cardiac cycle is correlated with duration of this cardiac cycle (CCD_0_), or with CCD occurring four (CCD_+4_), eight (CCD_+8_), or 12 (CCD_+12_) cardiac cycles later. All correlation coefficients are normalized to 1 at CCD_0_. Colors indicate units showing significant positive (pink) and negative (green) correlation between firing rate and CCD_0_.

So far, we have analyzed neural firing within the current cardiac cycle. We further investigated whether the link between spontaneous firing rate and CCD extends over a few cycles only, indicating a vagally mediated process, or over longer time periods, compatible with sympathetic influences. This analysis was performed in the subset of four patients from whom we recorded continuous segments of data of ≥5 min, i.e., the data length recommended to properly evaluate fluctuations in heart rate (Task Force of the European Society of Cardiology and the North American Society of Pacing and Electrophysiology, 1996). In those four patients, 10 cells showed a significant CCD effect (six R+, four R−). We first computed the correlation between firing rate in a given cardiac cycle and the duration of subsequent cardiac cycles. As can be seen in Figure 5 in six cells, located either in the MTL or the cingulate cortex, the correlation was maximal for the current CCD, and decreased over time, a pattern suggesting vagal influences. In the remaining four cells, all located in the MTL, the correlation increased after a few cycles and was maintained for much longer durations, ≤12 cardiac cycles later, compatible with either sympathetic or vagal influences.

**Figure 6.**
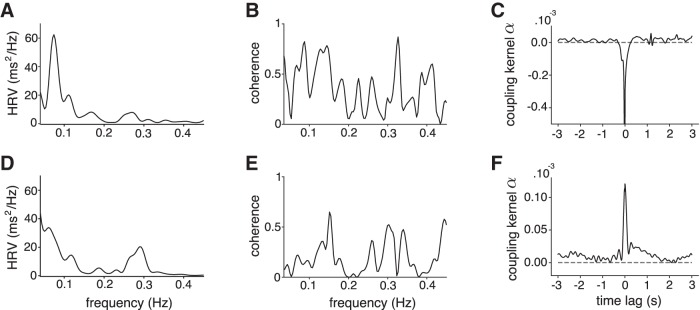
***A***, ***D***, Power spectrum of HRV in patient #6 (***A***) and patient #12 (***D***). ***B***, ***E***, Coherence between HRV and firing rate in two units. No distinctive peak corresponding to high-frequency or low-frequency HRV could be observed. ***C***, ***F***, Coupling kernel (α) between the smoothed ISI and CCD time series for two units.

In the same 10 cells, we then investigated whether the correlation between firing rate and CCD was modulated by respiration, as observed in the cat, in somatosensory thalamic neurons (Massimini et al., 2000). Indeed, CCD typically decreases during inspiration and increases during expiration. This modulation can be quantified by the spectral analysis of fluctuations in CCD over time, also known as HRV, where a peak between 0.15 and 0.4 Hz captures cardiac-rate changes locked to respiration. All patients showed normal HRV spectra (Fig. 6*A*,*D*), but the coherence between firing rate and HRV spectra did not reveal any distinctive peak in either the low-frequency or high-frequency range in any of the 10 cells analyzed (Fig. 6*B,E*). The coupling between firing rate and CCD that extends over several cycles is thus not related to respiration.

### Fast, beat-to-beat fluctuations

The analysis across several cycles shows that the coupling between firing rate and CCD can persist over several seconds, but that it is not linked to cardiorespiratory modulation. To test whether the coupling also has a fast component related to beat-to-beat changes that would be a hallmark of vagal influences, we used a different method to assess short-range temporal correlations between variations in firing rate and CCD. In the 25 neurons showing a significant correlation, we extracted the effective coupling kernel between smoothed ISI and CCD time series, which is independent from the autocorrelation structure of each time series. All coupling kernels showed a marked narrow peak at zero time lag and very limited slower modulations (Fig. 6*C,F*). The interaction between the two time series occurred at a fast time scale, suggesting that the link between firing rate and CCD is partly explained, at least in part, by short-lived, beat-to-beat fluctuations.

### Directionality of the interaction between firing rate and CCD

The correlation between firing rate and CCD does not inform us about the directionality of the effect. We first searched for evidence of directed interactions going from the heart to spiking activity by testing whether heartbeats trigger a transient change in firing rate. We analyzed the spike-density function locked to heartbeats and tested whether a heartbeat would trigger a transient increase or decrease in firing rate in any of the 25 neurons with a significant CCD effect. Of the 25 neurons, only two showed a transient increase in firing rate and one showed a transient decrease. None of those observations survived correction for multiple comparisons. We then used a model-based approach to test whether heartbeats elicit transient changes of neuronal firing activity. Each cell was described by a simple spiking neuron model with noisy background inputs and an additional input current triggered by heartbeats. Parameters were fitted using the observed spike trains and heartbeat times (Ladenbauer et al., 2018). Significance was assessed using a permutation-based procedure based on shuffled data. None of the 25 neurons showed a significant modulation of firing rate caused by heartbeats, according to this analysis. Thus, there is little evidence that heartbeats trigger a transient neural response in the 25 neurons showing a CCD effect.

**Figure 7.**
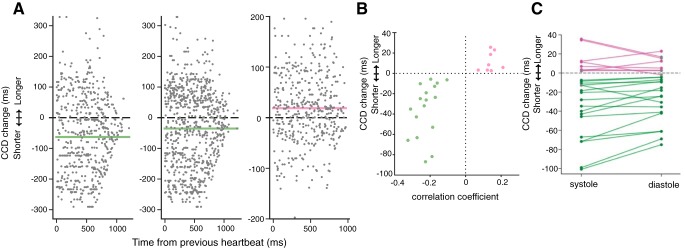
Effects of spikes and their timing on CCD. ***A***, Change of current CCD caused by spikes depending on their timing since the last heartbeat compared with the expected CCD (gray dots) and mean impact on CCD averaged across all spikes regardless of their timing (green line for shortening, pink line for lengthening) for three example units. ***B***, Mean impact on CCD versus correlation coefficient between firing rate and CCD for the 25 units with significant correlation. ***C***, Average change of CCD caused by spikes during systole and diastole separately for the 25 units from ***B*** (green or pink dots indicate a significant difference from 0). Lines connect average impact on CCD by unit. The influence of spikes on CCD was larger during systole in 20 of 25 units.

We then searched for evidence of directed interactions in the other direction, i.e., an influence of neural firing on CCD. To test whether the spiking activity of any of the 25 significant neurons exerts an effect on CCD, we used a phase–response analysis that quantifies how much cardiac cycles are shortened or lengthened depending on the occurrence and timing of spikes within the cardiac cycle (Fig. 7*A*; see Materials and Methods). In all 25 neurons, we found that neuronal spikes were significantly associated with a lengthening or shortening of the cardiac cycle (all FDR-corrected Monte-Carlo *p*'s < 0.05). Depending on neurons, the mean cardiac-cycle shortening or lengthening induced by neural firing varied from −87.2 to +25.3 ms (Fig. 7*B*). Cardiac-cycle lengthening or shortening revealed by the phase–response analysis was directly related to the strength and sign of the correlation between firing rate and CCD (Fig. 7*B*; correlation between cardiac-cycle lengthening and Pearson correlation coefficient between firing rate and CCD, Pearson *r* = 0.75, *p* < 10^−4^). The correlation between firing rate and CCD can thus mostly be accounted for by the influence of individual spikes on CCD.

Because of transmission delays between the recorded structures and the heart, spikes occurring early in the cardiac cycle (systole) should have more influence on the timing of the next heartbeat than spikes occurring late in the cardiac cycle (diastole). We found that it was indeed the case (Fig. 7*C*) in 20 of 25 neurons, with a significantly larger influence of spikes in systole than in diastole (paired *t* test, *t*_(24)_ = 3.13, *p* = 0.0045). Altogether, these results speak in favor of a directionality of interaction flowing from spiking activity to the heart.

Last, we found that R+ and R− neurons could be observed in the same region, from microwires only a few millimeters apart. We tested whether colocalized R+ and R− neurons systematically display a negative correlation between their firing rate, i.e., whether when a R− neuron fires and shortens the cardiac cycle, the R+ neuron remains relatively quiet. The data do not speak in favor of such a simple mechanism. We correlated the firing rate per cardiac cycle in the four pairs of (R+, R−) neurons available in the recordings (Table 1) and observed all possible patterns: two negative correlations (one nonsignificant; one significant *p* = 0.0026 uncorrected), two positive correlations (one nonsignificant; one significant *p* = 0.0007 uncorrected).

## Discussion

We recorded single-unit and multiunit activity in humans and found that spontaneous firing rate is directly related to CCD in more than a third of the neurons in the anterior parahippocampal gyrus (36%, 18 of 50), a region that encompasses both entorhinal and perirhinal cortices. We observed the same phenomenon, but in smaller proportion, in the mid-to-anterior cingulate cortex (11%, five of 45), and verified that this effect was not due to cardiac-related recording instability. Up to 11% of the variance of spontaneous firing rates was linked to fast, beat-to-beat changes in CCD, and neurons with a significant correlation between firing rate and CCD displayed an increased temporal variability in mean firing rate. The analysis of directed interactions pointed toward a direction of information flow from neurons to the heart. The detailed analysis of the temporal delays in neuron–heart coupling further reveals the involvement of a vagally mediated influence.

### CCD and firing rate in anterior parahippocampal regions and cingulate cortex

The regions where we find the correlation between firing rate and CCD are known to be related to HRV. Both cingulate regions (Ter Horst et al., 1996) and the amygdala and surrounding parahippocampal structures (Price and Amaral, 1981; Schwaber et al., 1982) project to autonomic nuclei, and the electrical stimulation of those regions alters CCD both in monkeys (Smith, 1945) and humans (Selimbeyoglu and Parvizi, 2010). Moreover, the BOLD signal in both cingulate and MTL regions fluctuates with HRV during tasks (Thayer et al., 2012; Beissner et al., 2013) as well as during resting state (Chang et al., 2013; Rebollo et al., 2018). Our results further show that the link between neural activity and CCD in both cingulate and medial temporal regions is directly reflected in fluctuations of spontaneous firing rate.

Perhaps the most striking feature of our results is the very large prevalence (36%, 18 of 50) of cardiac-related units in the anterior part of the parahippocampal gyrus, confirming earlier findings (Frysinger and Harper, 1989). The anterior parahippocampal gyrus contains the entorhinal and perirhinal cortices, located at the interface between the neocortex and the hippocampus. These cortices play an important role in spatial navigation and memory (Fyhn et al., 2004). Neuronal loss in this region is associated with cognitive impairments (Braak and Braak, 1991; Gómez-Isla et al., 1996). Spatial navigation, a behavior that strongly engages the entorhinal cortex (Fyhn et al., 2004), could benefit from a fast and precise regulation of heart rate to anticipate, and react to, the metabolic demands of walking and running.

The data presented here come from epileptic patients, which raises the question of whether the present results extend to healthy organisms. Epileptic patients tend to show a moderate reduction in HRV (Sevcencu and Struijk, 2010) that may be related to medication or epilepsy (Tomson et al., 1998). The data analyzed here corresponded to a quiet resting state, devoid of any seizure. Several arguments suggest that the results presented here are not specific to epileptic patients. First, the regions where we find a link between firing rate and CCD show no systematic relationship with the seizure-onset zone. Second, as detailed above, the regions where we find the CCD effect are known to be involved in cardiovascular regulation, either in animals or healthy human participants. Last, while all patients were under medication at the time of recordings, there was no consistent pattern between the presence of any given drug and the presence of the CCD effect.

### Cortical influence on heart rate

We found that changes in firing rate influenced heart rate, with several characteristics suggesting a vagal pathway: (1) the correlation between firing rate at time *t* and subsequent CCDs broke down after a few cycles in a number of cells, (2) the coupling between firing and CCD included a fast component, acting within a single cardiac cycle, and (3) the directed interaction from the neuron to the heart was most pronounced at systole. Note that slower modulations could also be observed, with a link between firing rate and heart rate spanning several cycles. The slower mechanism could coexist with the fast, vagal influence in the same neuron. A putative anatomical pathway for such modulation relies on the projections of the parahippocampal areas (Pitkänen et al., 2000) and cingulate regions (Pandya et al., 1981) to the amygdala, which in turn projects directly to the vagal nuclei in the medulla, the nucleus ambiguus, and dorsal motor nucleus of the vagus nerve (Hopkins and Holstege, 1978; Schwaber et al., 1982; Spyer, 1994). Still, the analysis we performed cannot rule out the possibility that the link between firing rate and CCD is mediated by another structure, not investigated in the present dataset, that would influence both firing in entorhinal and cingulate cortices on the one hand, and CCD on the other. In line with previous results from Frysinger and Harper (1989), the correlation between firing rate and CCD did not appear to be linked to the respiratory-related changes in heart rate (Eckberg, 2009).

Our results further emphasize the colocalization of neurons that increases or decreases heart rate within the same region. This finding is in line with the observation that the stimulation of the central nucleus of the rat amygdala (Iwata et al., 1987) or of the monkey entorhinal cortex (Reis and Oliphant, 1964) can trigger either increases or decreases in heart rate. Similarly, in humans, both heart-rate increases and decreases are related to activity in the cingulate cortex, as measured with fMRI (Gianaros and Wager, 2015).

### A novel factor accounting for the spontaneous variations in firing rate

So far, spontaneous variations in firing rate have been attributed to cellular machinery noise (Faisal et al., 2008), to brain state (Poulet and Petersen, 2008; Deco and Hugues, 2012; McGinley et al., 2015), and to fluctuations in excitability (Gilbert and Sigman, 2007; Goris et al., 2014). Our results suggest that in the parahippocampal region, and to a lesser extent in the cingulate cortex, the fine tuning of the CCD represents a novel, physiological factor accounting for a non-negligible part of the spontaneous variance in firing rate.

Changes in heart rate are often thought to be confined to emotions, pain, stress, and physical effort. It is worth emphasizing that nonemotional standard tasks, such as visual or auditory detection tasks (Lacey and Lacey, 1978; Bradley, 2009; Park et al., 2014; Raimondo et al., 2017), are associated with a precise and reproducible change in CCD. The functional imaging literature in humans has stressed the convergence of cognitive function and cardiac regulation in both the cingulate cortex (Critchley et al., 2003) and in the amygdalohippocampal region (Gianaros et al., 2004). It remains to be determined whether the variance in firing rate related to cardiac-cycle regulation is detrimental, or beneficial (Harris and Wolpert, 1998; McDonnell and Ward, 2011) to the cognitive information processing carried out in the parahippocampal and cingulate regions.
